# Prognostic impact of long-term beta-blockers post-stemi: a meta-analytic review

**DOI:** 10.1097/MS9.0000000000004665

**Published:** 2026-09-14

**Authors:** Iqra Maryam, Sagreeka Raj, Sheela Bai, Fnu Muskan, Sanjana Devi, Fnu Geeta, Ghazi Uddin Ahmed, Areeba Pervaiz, Deepika Deepika, Sayed Jawad

**Affiliations:** a Department of Medicine, Fatima Jinnah Medical University, Lahore, Pakistan; b Ghulam Muhammad Maher Medical College, Sukkur, Pakistan; c Liaquat University of Medical and Health Sciences, Jamshoro, Pakistan; d Peoples University of Medical and Health Sciences, Nawabshah, Pakistan; e Indus Medical College, Tando Muhammad Khan, Pakistan; f Chandka Medical College, Larkhana, Pakistan; g Jinnah Sindh Medical University, Karachi, Pakistan; h Ziauddin University, Karachi, Pakistan; i University Hospitals Birmingham, Birmingham, Pakistan; j Kabul University of Medical Sciences Faculty of Medicine, Kabul, Afghanistan

## Abstract

**Aims:**

This meta-analysis assessed the long-term impact of beta-blocker therapy on all-cause mortality and cardiovascular outcomes in patients with ST-elevation myocardial infarction (STEMI) who continued beta-blocker use for a minimum of 1 year after hospital discharge.

**Methods:**

Electronic databases were searched through September 2024 to identify studies comparing long-term outcomes in patients treated with beta-blockers versus those untreated. A random-effects model was used to estimate Hazard Ratios (HRs) with 95% confidence intervals (CIs).

**Results:**

A total of 21 studies involving 270 566 patients were included. In pooled analyses, extended beta-blocker was associated with significantly lower all-cause mortality in STEMI patients [HR: 0.74 (0.66, 0.83); *P* < 0.0001] and showing non-significant trend toward reducing cardiac death [HR: 0.77 (0.56, 1.04); *P* = 0.09], an effect observed predominantly in observational studies. However, in randomized controlled trials (*N* = 3), no significant benefit was demonstrated (all-cause mortality: *P* = 0.67; cardiac death: *P* = 0.62). No meaningful differences were found for major adverse cardiovascular events [HR: 0.90 (0.76, 1.07); *P* = 0.22], myocardial infarction, hospitalization for heart failure, or revascularization.

**Conclusion:**

Long-term beta-blocker use is associated with a significant reduction in all-cause mortality among STEMI patients, though this benefit was not seen in RCTs. Effects on other cardiovascular outcomes remain uncertain, highlighting the need for further large-scale trials.

## Introduction

Prior to the advent of routine reperfusion strategies, randomized controlled trials (RCTs) and meta-analyses demonstrated that beta-blocker therapy in patients with ST-elevation myocardial infarction (STEMI) was associated with improved clinical outcomes, including reductions in mortality and infarct size^[^1–3^]^. These benefits were primarily attributed to two core physiological mechanisms of beta-blockers: the reduction of myocardial oxygen demand and the augmentation of myocardial oxygen supply. Based on this evidence, current ACCF/AHA guidelines recommend the use of beta-blockers following STEMI, including in patients with complications such as heart failure (HF)[4]. However, these recommendations have drawn criticism due to their reliance on evidence derived from the pre-reperfusion era, raising concerns about their applicability in modern clinical practice.

HIGHLIGHTS

- Long-term beta-blocker use post-STEMI significantly reduces all-cause mortality in observational studies.
- No significant mortality benefit was observed in randomized controlled trials.
- Findings question the routine use of beta-blockers in patients with uncomplicated STEMI in the reperfusion era.

In the contemporary reperfusion era, immediate restoration of coronary perfusion via percutaneous coronary intervention (PCI) or fibrinolysis has become the standard of care for STEMI management. Alongside the development of evidence-based pharmacotherapies – such as antiplatelet agents, statins, and renin-angiotensin-aldosterone system inhibitors – the continued role of beta-blockers is increasingly questioned[5]. This issue is particularly relevant in light of growing polypharmacy, which increases the risk of adverse drug interactions and undermines patient adherence[6].

Furthermore, existing ACCF/AHA and international guidelines do not provide explicit recommendations regarding the optimal duration of beta-blocker therapy, contributing to ongoing uncertainty in clinical decision-making^[^4,7,8^]^. Additionally, recent studies have raised questions about the universal application of beta-blockers, suggesting that their effectiveness may vary based on the clinical characteristics of the STEMI presentation[9].

Findings from individual studies evaluating beta-blocker use in STEMI have been inconsistent, with some demonstrating benefit and others showing no significant effect^[^10–13^]^. A recent meta-analysis that aggregated these data was limited by the exclusion of RCTs, resulting in inadequate statistical power and reduced reliability[14]. Consequently, the present systematic review and meta-analysis aim to provide a robust, well-powered evaluation of the long-term impact of beta-blocker therapy in STEMI patients with preserved left ventricular systolic function. In preparing this manuscript, we have adhered to the TITAN Guidelines 2025 on the declaration and responsible use of artificial intelligence in academic publishing[15]. These standards ensure transparency, reproducibility, and integrity in the reporting process.

## Methods

This systematic review and meta-analysis were conducted in accordance with Cochrane guidelines and were reported following the Preferred Reporting Items for Systematic Reviews and Meta-Analyses (PRISMA) standards.

### Objectives, eligibility criteria, and outcomes

Studies were eligible for inclusion if they met the following criteria: (1) enrolled patients diagnosed with STEMI; (2) compared a beta-blocker with either placebo or no beta-blocker treatment; (3) reported at least one relevant outcome, including all-cause mortality, cardiovascular death, revascularization, myocardial infarction (MI), hospitalization for HHF, or major adverse cardiovascular events (MACEs); and (4) a minimum follow-up of 1 year after discharge was applied to all included studies; no exceptions were made. Studies including patients without HF or with a baseline left ventricular ejection fraction (LVEF) ≥ 40% were accepted.

Exclusion criteria comprised studies lacking adjusted comparative estimates between beta-blocker and no beta-blocker groups, as well as reviews, abstracts, editorials, case reports, and case series.

### Data sources and search

A comprehensive literature search was performed using the PubMed, Cochrane, and Scopus databases to identify studies published from their inception through September 2024. There were no restrictions on publication date, and no language restrictions were applied during the search; however, non-English articles were excluded due to feasibility constraints. The detailed search strategies applied for each database are provided in Supplemental Digital Content Table S1, available at: https://links.lww.com/MS9/B379. Additionally, both generic and brand names of all beta-blockers were used to search ClinicalTrials.gov for further eligible trials. Reference lists of identified studies, previous meta-analyses, and review articles were also screened to capture any additional relevant publications. When overlapping populations from registries were suspected, we planned to include the most complete or largest dataset to minimize duplication bias. Following the database searches, two independent reviewers (EA and SAW) screened titles and abstracts against predefined inclusion and exclusion criteria, and discrepancies were resolved by consensus. Full texts of potentially eligible studies were then examined to confirm final inclusion.

### Data extraction and quality assessment

Data extracted from eligible studies included trial characteristics, baseline patient demographics, safety information, and relevant outcomes. When additional information was required, corresponding authors were contacted. The quality of randomized trials was assessed using the Cochrane Risk of Bias tool[16], whereas observational studies were appraised with the Newcastle-Ottawa Scale[17]. Two independent reviewers (EA and SAW) performed these quality assessments, with any discrepancies resolved through consensus discussion.

### Statistical analysis

To assess the effect of beta-blockers, hazard ratios (HRs) were pooled across studies. The natural logarithm of each HR was calculated and combined using the inverse-variance method. Standard errors were derived from the confidence intervals reported in each study. Although most studies provided HRs, some reported only risk ratios (RRs); in these cases, RRs were used as approximations of HRs. Between-study heterogeneity was quantified using Higgins’ *I*^2^ statistic, which indicates the percentage of variation attributable to heterogeneity. A random-effects model was applied to generate pooled HR estimates, accommodating variability among studies. Forest plots were used to display pooled results, while funnel plots assessed potential publication bias. Subgroup analyses compared results from RCTs with those from observational studies. Sensitivity analyses were conducted by omitting each study sequentially to evaluate its impact. Statistical significance was set at *P* < 0.05. All analyses were conducted using RevMan version 5.4 (Cochrane Collaboration, Oxford, UK).

## Results

### Literature search and selection

The PRISMA flow diagram (Supplemental Digital Content Figure S1, available at: https://links.lww.com/MS9/B379) summarizes the study selection process. Following the initial screening of 9351 titles and abstracts, 29 articles were retrieved for full-text review. Ultimately, 21 studies evaluating long-term beta-blocker therapy in STEMI patients, encompassing a total of 270 566 participants, satisfied the inclusion criteria and were incorporated into the meta-analysis. Of these, 3 were RCTs^[^18–20^]^, while the remaining 18 were observational studies^[^9,10,12,21–35^]^.

### Study characteristics

Across the included studies, patient demographics and clinical characteristics showed notable variability. Mean age ranged from 56 ± 12 to 68 ± 14 years, while the proportion of male participants ranged from 72.1% to 87% (Table 1). Reported average BMI values varied between 23.3 ± 3.0 kg/m^2^ and 27.9 ± 3.9 kg/m^2^. LVEF means ranged from 43.0 ± 11.0% to 58.1 ± 8.6%. The prevalence of prior MI ranged from 2.3% to 11.7%, hypertension from 22.3% to 85.9%, and diabetes mellitus from 10% to 41%. Duration of follow-up across studies extended from 1 to 9 years. Among the three RCTs, Watanabe *et al*[19] was assessed as high risk of bias, whereas the other two had moderate risk^[^18,20^]^. According to the Newcastle-Ottawa Scale, all but one observational study were deemed high quality: nine scored 8/9^[^10,12,29–35^]^, eight scored 7/9^[^9,21–27^]^, and El Nasara *et al* received 6/9, indicating moderate quality[28].

**Table 1:** Baseline characteristics.

Author	Sample Size (*n*), (beta blocker/no beta blocker)	Type of study	Method of adjusting confounder	Age years, mean (beta blocker/no beta blocker)	LVEF %	Follow-up time (years)
**Bao 2012**	3692 (1614/2078)	Retrospective Cohort Study	Multivariate	65.8/68.0	>50	2.6
**Choo 2014**	1754 (1412/342)	Retrospective Cohort Study	Propensity Score Adjusted	NA	>40	3
**D’ascenzo 2018**	3234 (1889/1345)	Retrospective Cohort Study	Propensity Score Adjusted	NA	>50	1
**Dondo 2017**	91 895 (88 542/3353)	Prospective Cohort study	Propensity Score Adjusted	NA	>30	1
**El Nasasra 2020**	7392 (6007/1385)	Prospective Cohort Study	Multivariate	NA	>40	9
**Engelbertz 2023**	128 319 (36 738/91 581)	Retrospective Cohort Study	Multivariate	NA	NA	3.4
**Fabris 2021**	433 (355/78)	Retrospective cohort study	Propensity Score Adjusted	NA	NA	1
**Ferreira 2020**	3145 (2526/619)	Prospective Cohort Study	Multivariate	62/68	>40	1
**Joo 2020**	5805 (5065/740)	Retrospective Cohort Study	Propensity Score Adjusted	NA	NA	4.7
**Konishi 2016**	424 (197/227)	Retrospective Cohort Study	Propensity Score Adjusted	64.0/64.2	>40	4.5
**Lee 2015**	901 (598/303)	Retrospective Cohort Study	Multivariate	56/61	>50	3.9
**Lopez 2020**	460 (417/43)	Retrospective Cohort Study	Multivariate	NA	>40	4.5
**Luca 2003**	1513 (1325/188)	Prospective Cohort Study	Multivariate	60/61	>40	3.9
**Nakatani 2013**	5628 (2880/2748)	Retrospective Cohort Study	Propensity Score Adjusted	64.7/64.7	NA	1
**Ozasa 2010**	910 (349/561)	Retrospective Cohort Study	Propensity Score adjusted	66.4/68.0	>40	1
**Park 2021**	2236	Retrospective Cohort Study	Propensity Score Adjusted	NA	NA	1
**Podlesnikar 2020**	270 (139/131)	Randomized Control Trial	NA	58.7/58.2	NA	5
**Roolvink 2017**	684 (336/347)	Randomized Control Trial	NA	62.4/62.5	>50	1
**Wang 2019**	6474 (2465/4009)	Retrospective cohort study	Multivariate	NA	NA	8
**Watanabe 2018**	794 (394/400)	Randomized Control Trial	NA	63.9/64.5	>50	3

### Results of the meta-analysis

#### All-cause death

Nineteen studies reported data on all-cause mortality. The pooled analysis demonstrated that extended beta-blocker therapy significantly reduced the risk of all-cause death in patients with STEMI [HR: 0.74 (0.66, 0.83); *P* < 0.00001; *I*^2^ = 77%] (Fig. 1).

**Figure 1.**
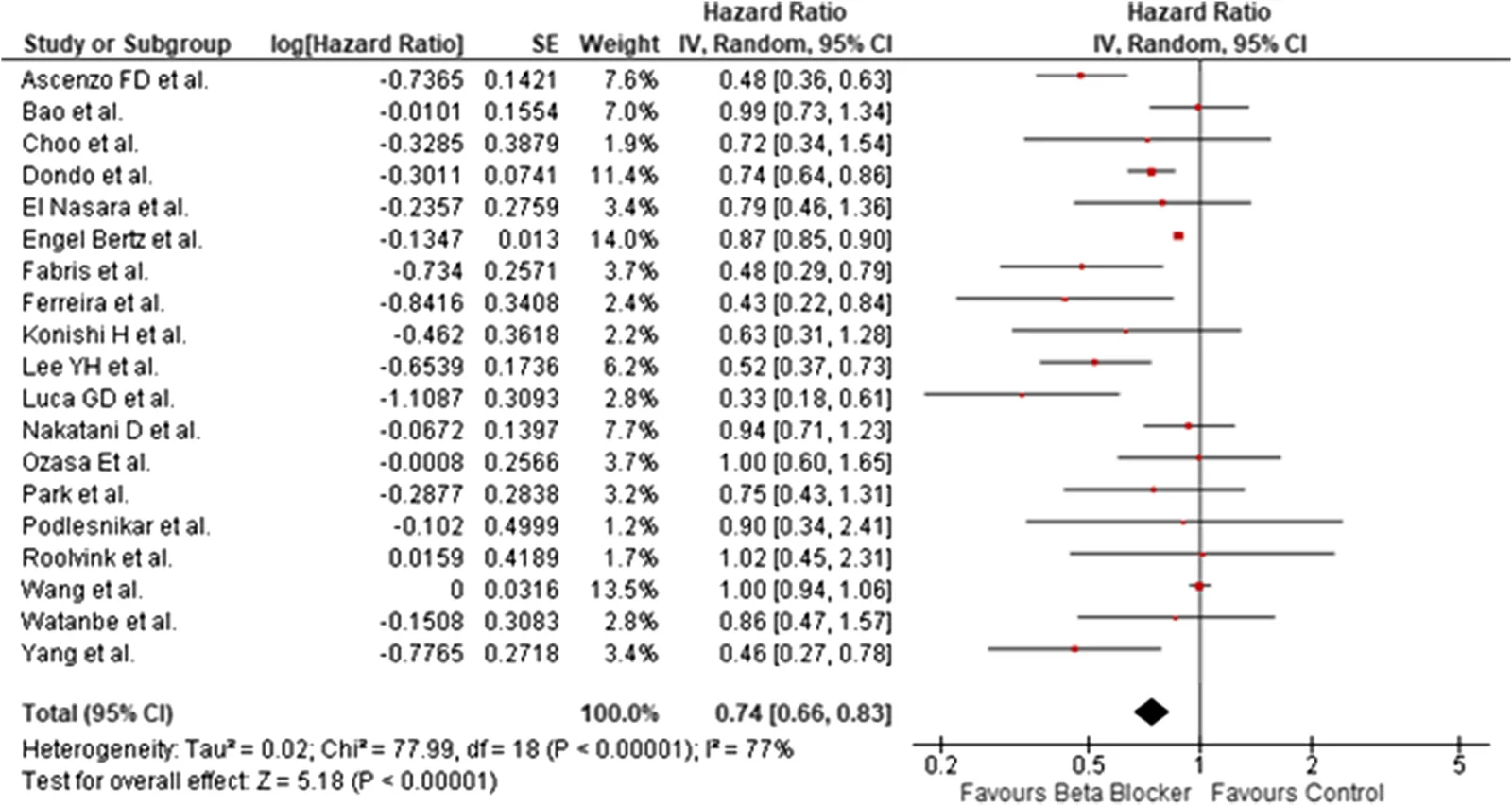
Forest plot for all-cause mortality.

#### Cardiac death

Ten studies assessed cardiac mortality. Meta-analysis indicated a non-significant trend toward reduced cardiac death in patients receiving long-term beta-blockers [HR: 0.77 (0.56, 1.04); *P* = 0.09; I^2^ = 37%] (Fig. 2).

**Figure 2.**
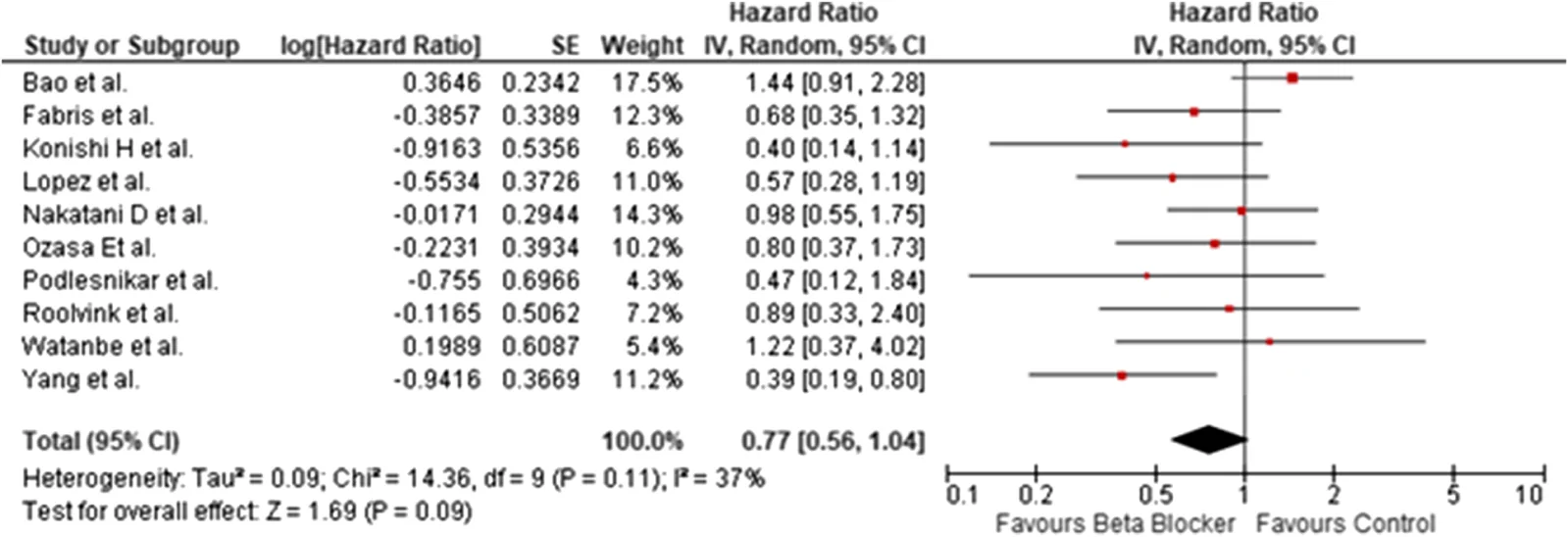
Forest plot of cardiac death.

#### Major adverse cardiovascular events

MACE were reported in nine studies. Pooled results indicated that extended beta-blocker therapy did not significantly alter the incidence of MACE in patients with STEMI [HR: 0.90 (0.76, 1.07); *P* = 0.22; I^2^ = 53%] (Fig. 3).

**Figure 3.**
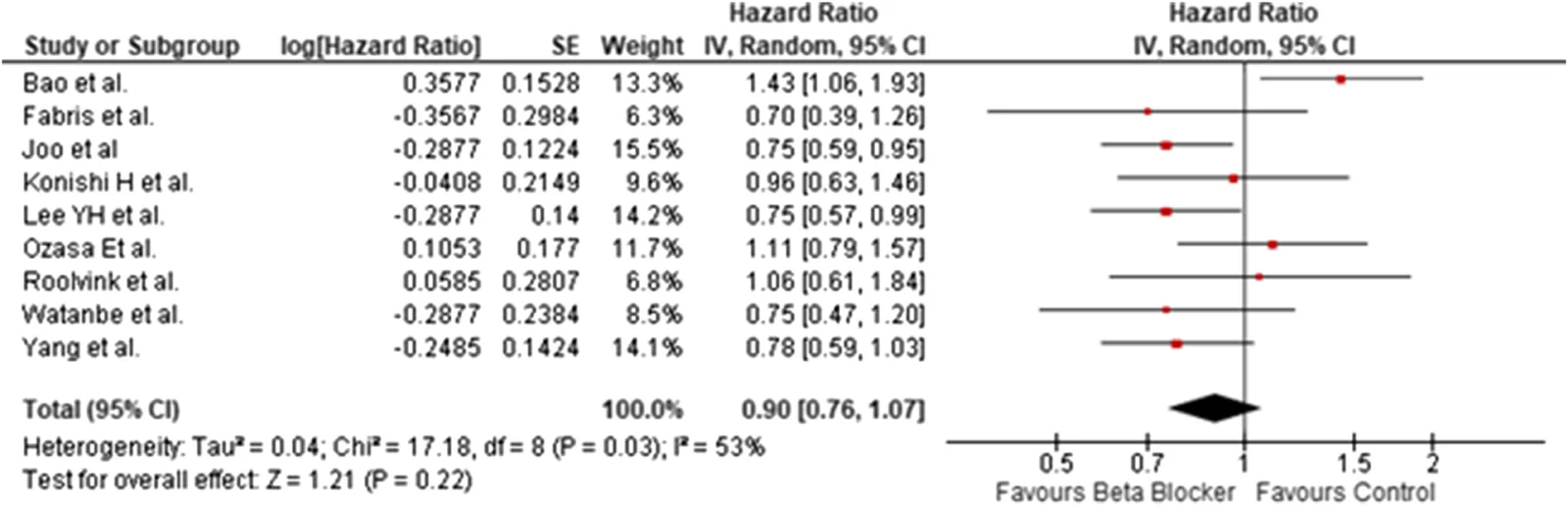
Forest plot for MACE.

#### Myocardial infarction

Six studies reported MI outcomes. Meta-analysis revealed no significant effect of prolonged beta-blocker therapy on MI incidence in STEMI patients [HR: 0.94 (0.57, 1.53); *P* = 0.79; *I*^2^ = 45%] (Fig. 4).

**Figure 4.**
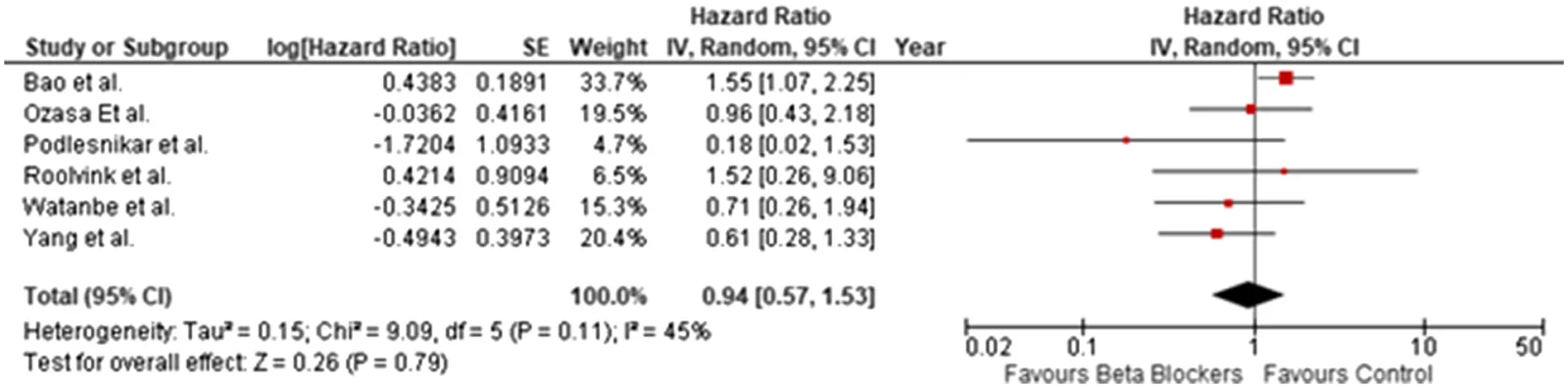
Forest plot for MI.

#### Hospitalizations for heart failure

Hospitalizations for HF (HHF) were reported in five studies. The meta-analysis demonstrated that prolonged beta-blocker therapy did not significantly affect the risk of HHF among STEMI patients [HR: 0.98 (0.56, 1.71); *P* = 0.93; I^2^ = 65%] (Fig. 5).

**Figure 5.**
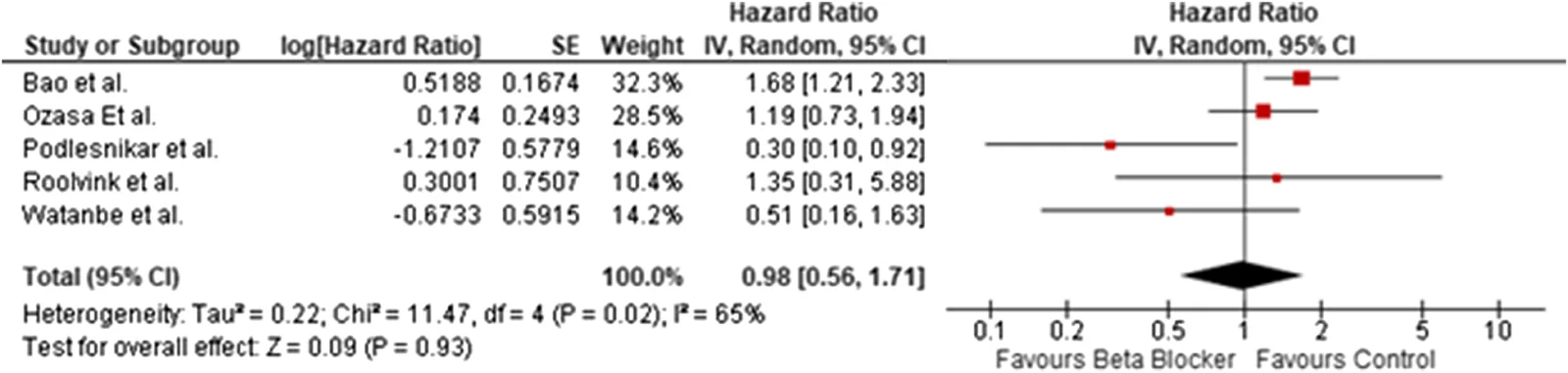
Forest plot of hospitalization for heart failure.

#### Revascularization

Revascularization outcomes were reported in three studies. Meta-analysis revealed that extended beta-blocker therapy had no significant effect on the incidence of revascularization in STEMI patients [HR: 1.00 (0.83, 1.20); *P* = 0.97; I^2^ = 0%] (Fig. 6).

**Figure 6.**
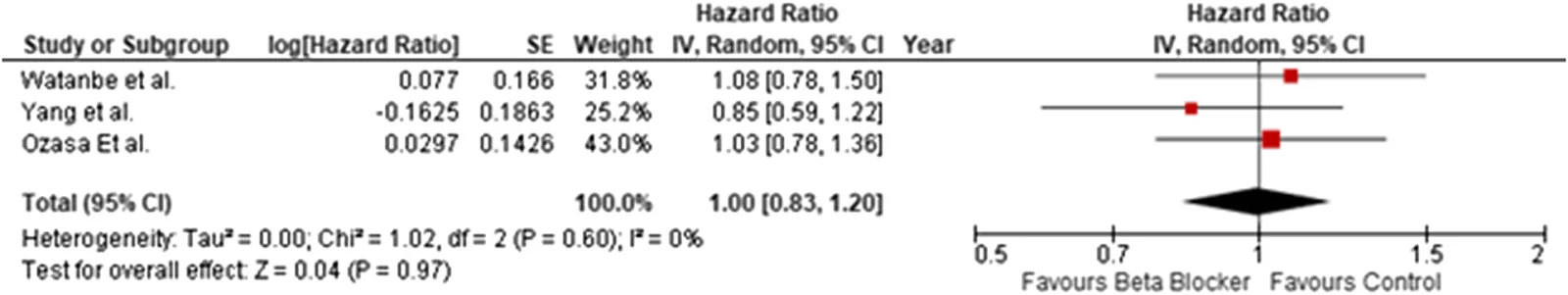
Forest plot for revascularization.

#### Sensitivity analysis

To evaluate the robustness of the findings, sensitivity analyses were conducted by sequentially excluding individual studies. Exclusion of Bao et al. significantly altered effect estimates and eliminated heterogeneity for both cardiac death [HR: 0.68 (0.52, 0.89); *P* = 0.005; *I*^2^ = 0%] and MACE [HR: 0.82 (0.73, 0.93); *P* = 0.001; *I*^2^ = 0%] (Supplemental Digital Content Figure S2, available at: https://links.lww.com/MS9/B379). For MI, removing Bao et al. also resolved heterogeneity, but did not significantly affect the pooled estimate [HR: 0.73 (0.46, 1.16); *P* = 0.19; *I*^2^ = 0%] (Supplemental Digital Content Figure S3, available at: https://links.lww.com/MS9/B379). The findings of Bao et al. (a Japanese cohort of STEMI patients undergoing PCI) diverged from other included studies, likely due to younger patient age, higher prevalence of baseline comorbidities, and differences in study design and follow-up methodology. The smaller sample size may also have limited statistical power. Exclusion of Podlesnikar et al. led to a reduction in heterogeneity for HHF, without changing the direction or significance of the effect [HR: 1.31 (0.89, 1.93); *P* = 0.18; *I*^2^ = 34%] (Supplemental Digital Content Figure S4, available at: https://links.lww.com/MS9/B379). Sensitivity analysis for all-cause mortality revealed stable results, with no material change in heterogeneity or overall effect size.

#### Subgroup analysis by study design

Subgroup analyses were performed to assess whether outcomes varied by study design. In pooled analyses of three RCTs, long-term beta-blocker use was not associated with significant differences in the risk of all-cause death [HR: 0.91 (0.59, 1.41); *P* = 0.67; *I*^2^ = 0%] (Supplemental Digital Content Figure S5, available at: https://links.lww.com/MS9/B379), cardiac death [HR: 0.84 (0.43, 1.64); *P* = 0.62; *I*^2^ = 0%] (Supplemental Digital Content Figure S6, available at: https://links.lww.com/MS9/B379), MI [HR: 0.67 (0.27, 1.67); *P* = 0.40; *I*^2^ = 12%] (Supplemental Digital Content Figure S7, available at: https://links.lww.com/MS9/B379), or HHF [HR: 0.53 (0.24, 1.19); *P* = 0.12; *I*^2^ = 21%] (Supplemental Digital Content Figure S8, available at: https://links.lww.com/MS9/B379). Similarly, an analysis of the two RCTs reporting MACE showed no significant benefit [HR: 0.87 (0.61, 1.24); *P* = 0.43; *I*^2^ = 0%] (Supplemental Digital Content Figure S9, available at: https://links.lww.com/MS9/B379). Subgroup analysis for revascularization was not feasible, as only one RCT reported this outcome.

In contrast, analysis of sixteen observational studies demonstrated a statistically significant association between long-term beta-blocker use and reduced all-cause mortality [HR: 0.73 (0.65, 0.82); *P* < 0.00001; *I*^2^ = 81%] (Supplemental Digital Content Figure S10, available at: https://links.lww.com/MS9/B379). For all-cause mortality, substantial heterogeneity was observed. Subgroup analyses stratified by study design (RCTs vs. observational studies), follow-up duration, and geographic region did not materially reduce heterogeneity. However, for other outcomes, no significant associations were observed in observational studies: cardiac death [HR: 0.74 (0.50, 1.08); *P* = 0.12; *I*^2^ = 55%], MI [HR: 1.05 (0.59, 1.88); *P* = 0.86; *I*^2^ = 60%], and MACE [HR: 0.90 (0.74, 1.11); *P* = 0.32; *I*^2^ = 63%]. Notably, in the two observational studies reporting HHF, long-term beta-blocker use was paradoxically associated with a higher risk of HHF [HR: 1.49 (1.07, 2.05); *P* = 0.02; *I*^2^ = 24%]. Overall, the apparent mortality benefit was largely driven by observational studies, whereas RCTs alone did not demonstrate a significant effect.

## Discussion

In this meta-analysis comprising 270 566 patients with STEMI who underwent revascularization, we assessed the efficacy of long-term beta-blocker therapy. Importantly, the majority of included patients had preserved LVEF and no clinical HF at baseline. Our results indicate that while long-term beta-blocker use was linked to a significant reduction in all-cause mortality, this effect was confined to observational studies. No statistically significant reductions were observed in cardiac death, MACEs, recurrent MI, HHF, or the need for repeat revascularization. These findings suggest that routine, indefinite beta-blocker therapy may offer limited benefit in patients with uncomplicated STEMI following successful revascularization.

Previous meta-analyses have also suggested a mortality benefit from long-term beta-blocker therapy in STEMI patients with preserved systolic function following PCI. However, these earlier efforts had important methodological limitations. For instance, the study by Misumida *et al* included only six observational cohorts, while Maqsood *et al* relied heavily on non-randomized data, incorporating only a single RCT. This reliance raises concerns about inherent biases – particularly selection bias – in which patients who were not prescribed beta-blockers may have had contraindications such as bradycardia or hemodynamic instability, potentially exaggerating the perceived benefit. Additionally, both prior meta-analyses failed to evaluate key endpoints such as recurrent MI, HHF, cardiac mortality, and revascularization.

To address these gaps, our analysis included a broader spectrum of outcomes and incorporated two recently published RCTs. Although our pooled data showed a statistically significant reduction in all-cause mortality, sensitivity analysis (excluding Bao *et al*) revealed additional significance in outcomes such as MACE, MI, and HHF. Still, these findings were largely influenced by observational data, increasing the risk of type I error. Subgroup analysis by study type revealed that RCTs did not demonstrate statistically significant improvements in all-cause death, MACE, MI, or HHF, highlighting a disparity in results that raises questions about the reliability of previous conclusions based solely on observational evidence. The lack of effect in RCTs may also reflect limited statistical power, relatively short follow-up durations, and selective inclusion criteria, which together reduce the likelihood of detecting differences in mortality. A one-size-fits-all approach appears increasingly untenable. Instead, future research should investigate whether β-blocker therapy can be tailored based on infarct size, residual ischemia, ventricular remodeling, or autonomic tone, thereby aligning β-blocker use with the pathophysiology and risk profile of individual patients. This discrepancy reinforces concerns that earlier reported benefits may be overestimated due to confounding factors and larger sample sizes typical of non-randomized designs.

Currently, long-term beta-blocker therapy is endorsed by the 2013 American Heart Association (AHA) and 2017 European Society of Cardiology (ESC) STEMI guidelines. While the benefit of beta-blockers in post-MI patients with LVEF <40% is well established, the evidence for their continued use in those with preserved or near-normal systolic function is sparse. The 2023 AHA/ACC guidelines provide a Class IIb recommendation for discontinuing beta-blockers after 1 year in patients with LVEF ≤50%, though this applies primarily to individuals with chronic coronary syndrome (CCS) rather than the acute STEMI setting. Therefore, the most up-to-date guidance specific to STEMI still relies on older recommendations from the pre-reperfusion era, when beta-blockers were tested in settings with less advanced adjunctive therapies and broader inclusion criteria, including patients with heart failure or ventricular arrhythmias. As modern management strategies, such as timely PCI, potent antithrombotic agents, and aggressive lipid-lowering therapies, have markedly improved prognosis and reduced mortality, the incremental benefit of beta-blockers in this contemporary context appears to have diminished.

Guideline recommendations for long-term beta-blocker use after STEMI largely stem from pre-reperfusion-era trials, where patients were at higher baseline risk and lacked access to contemporary reperfusion strategies and secondary prevention therapies. More recent evidence, including the REDUCE-AMI trial and the ABYSS study, suggests that in the modern reperfusion era, the benefits of chronic beta-blocker therapy are less pronounced in patients with preserved LVEF and uncomplicated clinical courses. These findings reinforce the need for an individualized approach rather than a universal, lifelong prescription strategy. From a clinical standpoint, the accumulated evidence supports continuing beta-blockers during the first year after STEMI, particularly given potential benefits on arrhythmia prevention and myocardial remodeling. However, in patients with preserved LVEF (>50%) and an uncomplicated post-MI course, discontinuation after 1 year appears reasonable and is supported by recent trial data. In contrast, patients with reduced LVEF, recurrent angina, arrhythmias, or other high-risk features may continue to derive benefit from longer-term therapy.

Our findings are consistent with recent trial data, including the REDUCE-AMI trial, which showed no added mortality or MI benefit with long-term beta-blocker use in post-MI patients with LVEF > 50%. Similarly, the ABYSS trial, which enrolled patients with LVEF > 40%, found noninferiority with discontinuation of beta-blockers, aside from a slight increase in cardiovascular hospitalizations, a result potentially biased by the open-label design. Importantly, neither trial found differences in hard outcomes such as mortality or recurrent MI. Collectively, these findings support a more individualized approach to beta-blocker therapy. In patients with uncomplicated STEMI, preserved LVEF, and complete revascularization, the routine long-term use of beta-blockers may be unnecessary. However, for patients with reduced LVEF, incomplete revascularization, or other complications, beta-blockers may still provide a protective advantage. A tailored strategy, guided by patient-specific clinical features, is therefore warranted to optimize treatment efficacy and avoid unnecessary pharmacotherapy.

### Limitations

Our meta-analysis has several important limitations that warrant consideration. First, although we conducted subgroup analyses of RCTs for all outcomes except revascularization, the number of available RCTs was small (*n* = 3), and the lack of statistically significant effects in these RCTs may therefore reflect limited statistical power rather than a true absence of benefit, which underscores the need for adequately powered randomized studies to clarify the causal impact of beta-blockers in this population. Despite efforts to adjust for confounders using multivariate analyses or propensity score matching, these methods cannot account for unmeasured confounding, which may have introduced bias; in particular, unmeasured comorbidities, differences in adherence to prescribed medications, socioeconomic factors, or variations in follow-up intensity could have contributed to the apparent survival benefit observed in observational cohorts, potentially inflating the magnitude of effect compared with randomized data. Second, substantial heterogeneity was observed across several outcomes, and while we performed sensitivity and subgroup analyses to identify potential sources, this heterogeneity remained unresolved in some cases, as we were unable to conduct formal meta-regression due to the limited number of included studies and insufficient reporting of study-level covariates; although subgroup analyses did not identify clear sources of heterogeneity, residual unexplained variability remains a limitation and may reflect unmeasured differences in populations, treatment protocols, or risk profiles across studies. Excluding Bao *et al* substantially altered the pooled estimate for cardiac death and MACE, and this study was identified as an outlier because of its shorter follow-up duration and inclusion of a higher-risk population compared with other cohorts, which may partly explain its disproportionate effect on the pooled estimates. Third, although most studies reported HRs, some did not, and in such cases, we approximated relative risks (RRs) as HRs, assuming equivalence, an assumption generally considered reasonable when event rates are low and evenly distributed between groups but one that may have compromised the precision of our pooled estimates in settings where this was not the case. Fourth, the definition of MACEs varied across studies, limiting comparability and likely contributing to heterogeneity, thereby affecting the generalizability of our findings. Fifth, we were unable to assess the impact of beta-blocker dosage due to the lack of dose-specific data, which may be relevant given the dose-dependent effects of beta-blockers. Sixth, although most included patients had preserved LVEF, stratified data by LVEF subgroups (e.g., 40–50% vs >50%) were rarely reported, and consequently we could not assess whether treatment effects differed across LVEF strata, which remains an important gap for future research. Another limitation is that the definition of MACEs was not uniform across included studies; while all definitions encompassed mortality and recurrent ischemic outcomes, some also included HF hospitalization or revascularization, and this variability may introduce heterogeneity and limit the comparability of pooled estimates. Moreover, the geographic distribution of included studies was skewed: most were conducted in high-income countries, with some from upper-middle-income settings and none from low- or lower-middle-income regions, which may limit the global generalizability of our findings. Finally, we could not evaluate the differential effects of individual doses and beta-blocker classes; while some studies reported the use of selective versus non-selective agents, subgroup data were insufficiently detailed and inconsistent across studies to allow reliable pooled analysis, and given that different agents may exert distinct pharmacologic effects, this limitation may have obscured important variations in treatment efficacy, underscoring the need for future studies to explore class-specific outcomes, as dose intensity and cardioselectivity may meaningfully influence outcomes–for example, higher doses or cardioselective agents may provide greater anti-ischemic effects, whereas non-selective agents could increase adverse events in certain populations– and the absence of dose- and class-specific data therefore represents an important limitation, potentially obscuring clinically relevant heterogeneity in treatment effects.

## Conclusion

In summary, this meta-analysis found no consistent survival benefit of long-term beta-blocker therapy in STEMI patients with preserved LVEF treated in the contemporary reperfusion era. While observational data suggest possible reductions in recurrent ischemic events, these findings may be influenced by residual confounding. Moving forward, adequately powered randomized trials are needed to clarify the true effect of long-term beta-blockers in this setting. Future research should also explore whether therapy can be tailored based on patient subgroups, dose or drug class, and comorbidity profiles. Such evidence may help refine guideline recommendations and ensure that beta-blocker therapy is targeted to patients most likely to derive meaningful benefit. Robust, large-scale RCTs with prolonged follow-up are needed to confirm these results, to better inform clinical decision-making, and to determine whether specific patient subgroups may still benefit.

## Ethical approval

Not applicable. This study did not involve primary data collection from human participants or animals.

## Consent

Not applicable.

## Sources of funding

This research received no specific grant from any funding agency in the public, commercial, or not-for-profit sectors.

## Author contributions

All authors contributed to the conception and design of the work, interpretation of the findings, drafting and/or critical revision of the manuscript, and approved the final version for publication.

## Conflicts of interest disclosure

The authors declare no conflicts of interest.

## Research registration unique identifying number (UIN)

Not applicable.

## Guarantor

Sayed Jawad.

## Provenance and peer review

Not commissioned, externally peer-reviewed.

## Data availability statement

No new data were generated or analyzed for this study.

## References

[1] Norwegian Multicenter Study Group. Timolol-induced reduction in mortality and reinfarction in patients surviving acute myocardial infarction. N Engl J Med 1981;304:801–07. 10.1056/NEJM198104023041401 7010157

[2] Hjalmarson A Herlitz J Holmberg S. The Göteborg metoprolol trial. Effects on mortality and morbidity in acute myocardial infarction. Circulation 1983;67:I26–32. 6342837

[3] Beta-Blocker Heart Attack Trial Research Group. A randomized trial of propranolol in patients with acute myocardial infarction: II. Morbidity Results. JAMA 1983;250:2814–19. doi:10.1001/jama.1983.03340200048027. 6358542

[4] Writing Committee Members*, O’gara PT Kushner FG Ascheim DD. 2013 ACCF/AHA guideline for the management of ST-elevation myocardial infarction. Circulation 2013;127:e362–e425. 10.1161/CIR.0b013e3182742cf6 23247304

[5] Joo SJ. Beta-blocker therapy in patients with acute myocardial infarction: not all patients need it. Acute Crit Care 2023;38:251. 10.4266/acc.2023.00955 PMC10497890 37652855

[6] Abbott JD Goldberger JJ. β-blocker therapy after myocardial infarction: a little goes a long way. J Am Heart Assoc 2023;12:e030867. 10.1161/JAHA.123.030867 PMC10492990 37493027

[7] Farzam K Jan A. Beta blockers. In: StatPearls. StatPearls Publishing; 2024. Accessed October 26, 2024. http://www.ncbi.nlm.nih.gov/books/NBK532906/30422501

[8] Ibanez B James S Agewall S. 2017 ESC guidelines for the management of acute myocardial infarction in patients presenting with ST-segment elevation: the task force for the management of acute myocardial infarction in patients presenting with ST-segment elevation of the European Society of Cardiology (ESC). Eur Heart J 2018;39:119–77. 10.1093/eurheartj/ehx393 28886621

[9] Ozasa N Kimura T Morimoto T. Lack of effect of oral beta-blocker therapy at discharge on long-term clinical outcomes of ST-segment elevation acute myocardial infarction after primary percutaneous coronary intervention. Am J Cardiol 2010;106:1225–33. 10.1016/j.amjcard.2010.06.048 21029817

[10] Yang JH Hahn JY Song YB. Association of beta-blocker therapy at discharge with clinical outcomes in patients with ST-segment elevation myocardial infarction undergoing primary percutaneous coronary intervention. JACC Cardiovasc Interv 2014;7:592–601. 10.1016/j.jcin.2013.12.206 24947717

[11] Kim J Kang D Park H. Long-term β-blocker therapy and clinical outcomes after acute myocardial infarction in patients without heart failure: nationwide cohort study. Eur Heart J 2020;41:3521–29. 10.1093/eurheartj/ehaa376 32542362

[12] Dondo TB Hall M West RM. β-blockers and mortality after acute myocardial infarction in patients without heart failure or ventricular dysfunction. J Am Coll Cardiol 2017;69:2710–20. 10.1016/j.jacc.2017.03.578 PMC5457288 28571635

[13] Holt A Blanche P Zareini B. Effect of long-term beta-blocker treatment following myocardial infarction among stable, optimally treated patients without heart failure in the reperfusion era: a Danish, nationwide cohort study. Eur Heart J 2021;42:907–14. 10.1093/eurheartj/ehaa1058 33428707

[14] Kim Y Byun S Kim HY. Long-term beta-blocker therapy after myocardial infarction without heart failure in the reperfusion era-systemic review and meta-analysis. J Cardiovasc Pharmacol 2022;79:650–54. 10.1097/FJC.0000000000001221 35058412

[15] TITAN Group, Agha RA Mathew G Rashid R. Transparency In The reporting of Artificial intelligence –the TITAN guideline. Prem J Sci 2025;10:100082.

[16] Higgins JPT Altman DG Gøtzsche PC. The cochrane collaboration’s tool for assessing risk of bias in randomised trials. BMJ 2011;343:d5928. doi:10.1136/bmj.d5928 PMC3196245 22008217

[17] Ottawa Hospital Research Institute. Accessed 20 August 2024. https://www.ohri.ca/programs/clinical_epidemiology/oxford.asp

[18] Roolvink V Ottervanger JP Ibáñez B. One-year clinical outcome of early administration of intravenous beta-blockers in patients with ST-segment elevation myocardial infarction before primary percutaneous coronary reperfusion. EuroIntervention J Eur Collab Work Group Interv Cardiol Eur Soc Cardiol 2018;14:688–91. 10.4244/EIJ-D-17-00776 29155386

[19] Watanabe H Ozasa N Morimoto T. Long-term use of carvedilol in patients with ST-segment elevation myocardial infarction treated with primary percutaneous coronary intervention. PloS One 2018;13:e0199347. 10.1371/journal.pone.0199347 PMC6112626 30153268

[20] Podlesnikar T Pizarro G Fernández-jiménez R. Five-year outcomes and prognostic value of feature-tracking cardiovascular magnetic resonance in patients receiving early prereperfusion metoprolol in acute myocardial infarction. Am J Cardiol 2020;133:39–47. 10.1016/j.amjcard.2020.07.037 32819681

[21] Luca GD de Boer MJ Ottervanger JP. Impact of beta-blocker therapy at discharge on long-term mortality after primary angioplasty for ST-segment elevation myocardial infarction. Am J Cardiol 2005;96:806–09. 10.1016/j.amjcard.2005.05.025 16169366

[22] D’ascenzo F Celentani D Brustio A. Association of beta-blockers with survival on patients presenting with ACS treated with PCI: a propensity score analysis from the BleeMACS registry. Am J Cardiovasc Drugs 2018;18:299–309. 10.1007/s40256-018-0273-4 29691803

[23] Wang WH Cheng CC Mar GY. Improving outcomes in chronic obstructive pulmonary disease by taking beta-blockers after acute myocardial infarction: a nationwide observational study. Heart Vessels 2019;34:1158–67. 10.1007/s00380-019-01341-0 30680495

[24] Ferreira JA Baptista RM Monteiro SR. Usefulness of universal beta-blocker therapy in patients after ST-elevation myocardial infarction. Medicine (Baltimore) 2021;100:e23987. 10.1097/MD.0000000000023987 PMC7837933 33545989

[25] Padilla López A Alós-almiñana M Peris JE. Secondary adherence to beta-blockers after ST-elevation myocardial infarction without ventricular dysfunction. Med Clínica Engl Ed 2020;155:242–48. 10.1016/j.medcli.2019.11.008 31932044

[26] Fabris E Pezzato A Gregorio C. STEMI and multivessel disease: medical therapy amplifies the benefit of complete myocardial revascularisation. Heart Lung Circ 2021;30:1846–53. 10.1016/j.hlc.2021.06.522 34393047

[27] Engelbertz C Feld J Makowski L. Contemporary secondary prevention in survivors of ST-elevation myocardial infarction with and without chronic kidney disease: a retrospective analysis. Clin Kidney J 2023;16:1947–56. 10.1093/ckj/sfad219 PMC10616503 37915929

[28] El Nasasra A Beigel R Klempfner R. Comparison of Outcomes with or without beta-blocker therapy after acute myocardial infarction in patients without heart failure or left ventricular systolic dysfunction (from the Acute Coronary Syndromes Israeli Survey [ACSIS]). Am J Cardiol 2021;143:1–6. 10.1016/j.amjcard.2020.12.044 33359228

[29] Bao B Ozasa N Morimoto T. β-Blocker therapy and cardiovascular outcomes in patients who have undergone percutaneous coronary intervention after ST-elevation myocardial infarction. Cardiovasc Interv Ther 2013;28:139–47. 10.1007/s12928-012-0137-9 23054967

[30] Nakatani D Sakata Y Suna S. Impact of beta blockade therapy on long-term mortality after ST-segment elevation acute myocardial infarction in the percutaneous coronary intervention era. Am J Cardiol 2013;111:457–64. 10.1016/j.amjcard.2012.10.026 23228922

[31] Choo EH Chang K Ahn Y. Benefit of β-blocker treatment for patients with acute myocardial infarction and preserved systolic function after percutaneous coronary intervention. Heart 2014;100:492–99. 10.1136/heartjnl-2013-305137 24395980

[32] Lee YH Park JS Tahk SJ. β-blocker therapy in the era of primary percutaneous intervention for ST elevation myocardial infarction. Cardiology 2015;132:91–100. 10.1159/000431077 26112078

[33] Konishi H Miyauchi K Kasai T. Long-term effect of β-blocker in ST-segment elevation myocardial infarction in patients with preserved left ventricular systolic function: a propensity analysis. Heart Vessels 2016;31:441–48. 10.1007/s00380-014-0624-2 25573259

[34] Joo SJ Kim SY Choi JH. Effect of beta-blocker therapy in patients with or without left ventricular systolic dysfunction after acute myocardial infarction. Eur Heart J - Cardiovasc Pharmacother 2021;7:475–82. 10.1093/ehjcvp/pvaa029 32289158

[35] Park CS Yang HM Ki YJ. Left ventricular ejection fraction 1 year after acute myocardial infarction identifies the benefits of the long-term use of β-blockers: analysis of data from the KAMIR-NIH registry. Circ Cardiovasc Interv 2021;14:e010159. 10.1161/CIRCINTERVENTIONS.120.010159 33877862

